# The skin microbiome in aging from ecological dysbiosis to molecular therapeutics

**DOI:** 10.20517/mrr.2026.42

**Published:** 2026-10-09

**Authors:** Yu Lei, Qianmei Liu, Qianjin Lu

**Affiliations:** ^1^Hospital for Skin Diseases, Institute of Dermatology, Chinese Academy of Medical Sciences & Peking Union Medical College, Nanjing 210042, Jiangsu, China.; ^2^Key Laboratory of Basic and Translational Research on Immune-Mediated Skin Diseases, Chinese Academy of Medical Sciences, Nanjing 210042, Jiangsu, China.; ^3^Jiangsu Provincial Key Laboratory of Dermatology, Nanjing 210042, Jiangsu, China.; ^#^These authors contributed equally to this work.

**Keywords:** Skin aging, skin microbiome, dysbiosis, anti-aging intervention

## Abstract

Skin aging is a progressive process driven by cumulative alterations in skin structure and function, resulting in a distinct clinical phenotype characterized by wrinkles, loss of elasticity, pigmentary changes, and impaired barrier integrity. Although intrinsic aging and environmental stressors are well-established contributors, the skin microbiome is increasingly recognized as an important regulator of cutaneous homeostasis through its roles in modulating immune responses, maintaining barrier function, and regulating metabolic homeostasis. Age-associated physiological changes in the host selectively reshape the composition and function of the skin microbiome, while the resulting microbial dysbiosis may, in turn, contribute to the development of skin aging phenotypes. Here, we summarize the mechanistic crosstalk between the aging host and the skin microbiome, with particular emphasis on how these bidirectional interactions contribute to the pathogenesis of geriatric dermatoses. We further evaluate emerging microbiome-based therapeutic strategies, ranging from ecological restoration to precision microbial modulation. Viewing skin aging through this ecological framework may provide a foundation for developing translational strategies aimed at promoting healthier skin aging.

## INTRODUCTION

The global shift toward an aging population has made extending health span a fundamental goal of modern medicine. As the body’s primary interface with the external environment, the skin exhibits some of the most visible manifestations of aging. Traditionally, skin aging has been attributed to intrinsic aging processes, such as cellular senescence, together with cumulative extrinsic damage from the exposome, particularly ultraviolet (UV) radiation^[1]^. These factors drive characteristic age-associated changes, including structural atrophy, barrier dysfunction, and delayed wound healing. However, this host-centric perspective is now expanding toward a holobiont framework, which recognizes the skin as a unified ecosystem in which the host and its resident microbiota exist in dynamic symbiosis^[2,3]^. Under homeostatic conditions, this microbial community contributes to immune regulation and barrier integrity through the continuous exchange of metabolites and immunomodulatory signals^[4]^. Although the gut-skin axis has been widely discussed as a systemic modulator of skin health, local interactions at the cutaneous surface play a more direct role in tissue physiology.

Recent evidence supports a bidirectional association between the skin and its microbiome. Age-related physiological shifts alter the skin environment, including pH and lipid composition, and are associated with changes in microbial composition, including the decline of dominant commensals and increased representation of potentially pathogenic taxa^[5]^. These age-associated microbial alterations may, in turn, contribute to aging-related skin phenotypes through effects on host inflammatory responses, cellular senescence, and tissue homeostasis^[6,7]^. In this review, we summarize the ecological shifts reshaping the skin microbiome and their mechanistic links to geriatric dermatoses, such as senile atopic dermatitis, chronic wounds, and cutaneous malignancies. We further evaluate the potential and limitations of leveraging microorganisms for anti-aging applications, ranging from ecological restoration to precision modulation. By integrating host aging with microbial ecology, this review aims to inform the development of novel therapeutic and preventive strategies for skin aging.

## THE CHANGING LANDSCAPE

Unlike the nutrient-rich and anaerobic environment of the gut, the skin constitutes a physiologically restrictive habitat defined by high salinity and low nutrient availability^[3]^. These physical and chemical constraints select for microorganisms adapted to specific skin niches^[8]^. However, aging progressively alters both the composition and function of this ecosystem. To understand the transition from adult stability to age-related dysbiosis, it is necessary to consider the enabling technologies, the chronological trajectory of these changes, and the underlying physiological drivers.

Methodological advances have significantly refined the understanding of how the skin microbiome changes during aging. Early studies using culture-dependent methods identified dominant genera^[9]^, but substantially underestimated the complexity of the cutaneous biosphere. The subsequent adoption of culture-independent sequencing substantially expanded the detectable diversity of the skin microbiome. In particular, 16S rRNA gene sequencing enabled broad taxonomic profiling across different cutaneous sites^[10,11]^, whereas shotgun metagenomics subsequently provided higher taxonomic resolution, including strain-level diversity^[12,13]^. Building on these genomic foundations, a recent skin metatranscriptomics study by Chia et al. underscores the value of RNA-based approaches for identifying transcriptionally active microorganisms and microbial functions in situ^[14]^. Beyond sequencing, the integration of fluorescence-based optical approaches with artificial intelligence (AI) offers a promising strategy for rapid microbiota analysis. Fluorescence spectrometric and imaging data combined with machine learning can support high-dimensional bacterial identification and microbiota classification^[15]^. Similarly, super-resolution fluorescence imaging coupled with AI analysis has achieved bacterial detection at the single-cell level. This approach distinguishes common skin bacterial species without time-consuming amplification or complex sample processing^[16]^. Collectively, these innovations have moved the field beyond descriptive associations by enabling increasingly detailed investigation of the spatial and functional interactions between the microbiome and skin aging.

The skin microbiome is dynamic, with community structure evolving from birth and acquiring distinct features across the human lifespan. Initial colonization is shaped by delivery mode, whereby vaginally delivered infants acquire a *Lactobacillus*-dominant profile. In contrast, those born by cesarean section are exposed to maternal skin and environmental microbes^[17]^. During late adolescence, increased sebum production drives the establishment of a stable adult microbiome dominated by lipophilic organisms^[18]^. Under homeostatic conditions, this mature ecosystem is dominated by four major bacterial phyla, namely, *Actinobacteria* (e.g., *Cutibacterium* and *Corynebacterium*), *Firmicutes* (e.g., *Staphylococcus* and *Streptococcus*), *Proteobacteria* (e.g., *Acinetobacter*), and *Bacteroidetes* (e.g., *Prevotella*), alongside fungal species of the genus *Malassezia*^[19,20]^*.* In this phase, *Cutibacterium acnes* (*C. acnes*) functions as an important commensal species that metabolizes triglycerides and produces short-chain fatty acids (SCFAs), which help maintain the acidic skin surface and limit the growth of potentially pathogenic microorganisms^[21]^. Beyond bacterial and fungal communities, the cutaneous virome represents an additional component of the skin ecosystem. In healthy adults, the skin virome is highly individualized and site-specific, with bacteriophages dynamically associated with their bacterial hosts and capable of shaping bacterial community structure through predation, lysogeny, and genetic exchange^[22,23]^.

In contrast to the relative stability of adulthood, the aging skin microbiome undergoes a distinct transformation. Multiple cohorts have reported increased α-diversity in older individuals, particularly at sebaceous sites such as the face^[24-27]^. Rather than indicating a healthier microbiome, this increase may reflect the loss of dominance by commensals such as *C. acnes* and a redistribution toward other taxa, including environmental transients and potentially opportunistic taxa such as *Corynebacterium minutissimum* and various *Proteobacteria*. This age-associated restructuring is further characterized by reduced temporal stability, greater inter-individual variability, and weakened site-specific community structure^[24]^. Age-associated changes are not restricted to bacterial communities, as the skin mycobiome also shows shifts in diversity and composition^[28]^. Although the skin phageome remains poorly characterized, several cutaneous polyomaviruses exhibit age-associated dynamics, with the prevalence and viral loads of Merkel cell polyomavirus (MCPyV) and human polyomaviruses (HPyV) 6 and 7 increasing with age^[29,30]^. These findings are summarized in Table 1.

**Table 1:** Summary of studies which examined age-associated changes in the skin microbiota

**Sample collection site**	**Population**	**Age group**	**Method**	**Microbiome alterations**	**Clinical and functional associations**	**Reference**
Forehead, forearms, palms	40 Chinese participants	0-20 (*n* = 10), 21-50 (*n* = 19), and 51-90 years (*n* = 11)	16S rRNA gene sequencing	Elderly: Lower relative abundance of *S. aureus*	N/A	[31]
Cheeks, forehead	30 Thai females	19-24 (healthy, *n* = 10), 19-24 (acne, *n* = 10), and 51-57 years (elderly, *n* = 10)	16S rRNA gene sequencing	Elderly: Enriched in *Firmicutes* Younger: Dominated by *Gemmatimonadetes, Planctomycetes*, and *Nitrospirae*	N/A	[32]
Scalp, forehead, cheeks, volar forearm	37 healthy Japanese women	21-37 (*n* = 18), and 60-76 years (*n* = 19)	16S rRNA gene sequencing	Elderly: Higher α-diversity. Reduced relative abundance of *Cutibacterium* with a concurrent enrichment of oral taxa	Microbiome diversification is largely affected by skin aging and oral bacterial colonization	[27]
Forehead	34 healthy Western European women	21-31 (*n* = 17), and 54-69 years (*n* = 17)	16S rRNA gene sequencing	Elderly: Higher α-diversity. Enrichment of *Corynebacterium* and *Proteobacteria*; depletion of *Cutibacterium* and *Actinobacteria*	N/A	[25]
Cheeks, forearm, upper back	50 Chinese participants	4-6, 11-13, 25-34, 37-53, and 62-74 years, each comprising 10 individuals	16S rRNA gene sequencing	Elderly: Higher species richness and diversity; loss of skin-site selectivity	N/A	[33]
Forehead, nose crease, scalp, forearm, oral epithelium	495 North American participants	9-78 years	16S rRNA gene sequencing	Age is the primary factor shaping the microbiome, driven by specific, mutually exclusive *Corynebacterium* OTUs	These specific *Corynebacterium* taxa are independently correlated with chronological age, wrinkles, and pigment spots	[34]
Cheeks	73 healthy Chinese women	25-35 (*n* = 48), and 56-63 years (*n* = 25)	16S rRNA gene sequencing	Elderly: Enrichment of *Proteobacteria* and *Actinobacteria* Younger: Enrichment of *Bacteroidetes* and *Firmicutes*	Metabolic shift from replication and repair pathways in younger to biodegradation pathways in elderly	[35]
Hands, forehead	1,975 skin samples (United States, *n* = 1,723; United Kingdom, *n* = 27; others)	18-90 years	16S rRNA gene sequencing	Negative correlation between age and anaerobic taxa (e.g., *Mycoplasma*, *Enterobacteriaceae*, and *Pasteurellaceae*)	Skin microbiome models predict chronological age with high accuracy (mean absolute error < 4 years)	[36]
Forehead, hands	73 healthy Korean females	10-29 (*n* = 24), 30-49 (*n* = 21), and 50-79 years (*n* = 28)	16S rRNA gene sequencing	Elderly: Higher α-diversity on the forehead, but stable on hands. Age-related shifts in commensals (*Streptococcus, Staphylococcus*, *Cutibacterium*, and *Corynebacterium*) observed at both sites	N/A	[26]
Cheeks, abdomen	80 healthy Chinese participants	3-7, 19-23, 37-42, and 65-74 years, each comprising 20 individuals	16S rRNA and ITS rDNA gene sequencing	Identification of 9 age-discriminatory taxa, including *Cyanobacteria, Staphylococcus, Cutibacterium, Lactobacillus, Corynebacterium, Streptococcus, Neisseria, Candida,* and *Malassezia*	Identification of 18 pathways, notably antibiotic biosynthesis, which may impact skin aging	[28]
Cheeks, forehead	51 healthy Korean participants	21-36 (*n* = 25), and 49-67 years (*n* = 26)	16S rRNA gene sequencing	Elderly: Higher α- diversity. Predominance of *Enhydrobacter* Younger: Higher abundance of *Lawsonella*	*Lawsonella*, *Staphylococcus*, and *Corynebacterium* correlate negatively with skin spots. *Staphylococcus* (esp. *S. aureus*) and *Lawsonella* correlate with barrier impairment (high TEWL, low moisture)	[37]
Forearm, buttock, facial skin	158 Caucasian females	20-24 (*n* = 32), 30-34 (*n* = 26), 40-44 (*n* = 25), 50-54 (*n* = 24), 60-64 (*n* = 25), and 70-74 years (*n* = 26)	16S rRNA gene sequencing	Elderly: Significant decrease in *Lactobacillus* and *Cutibacterium* abundance across all skin sites	Microbiome composition correlates with age-related decrease in sebocyte area and increases in NMFs, AMPs, and skin lipids	[38]
Cheeks	51 healthy Caucasian females	20-26 (*n* = 26), and 54-60 years (*n* = 25)	Shotgun metagenomics	Elderly: Enrichment of *S. epidermidis* and *Corynebacterium kroppenstedtii* Younger: Higher proportion of *C. acnes*	Enrichment of TCA cycle and biosynthesis pathways in elderly. Biophysical characteristics of the skin, notably the diffusion coefficient of collagen, correlate with both the composition and functional capabilities of the skin microbiome	[39]
	13 skin microbiome datasets	18-70 years	16S rRNA gene sequencing	Elderly: Higher α-diversity. Decreasing trend in *Cutibacterium* abundance	Lower grades associate with commensals (*Staphylococcus*, *Kocuria*, *Peptostreptococcus*, and *Lysobacter*); higher wrinkle grades correlate with *Brevibacterium* and *Kaistella*	[40]
Facial skin	100 healthy female Caucasian volunteers	18-35 (*n* = 50), and 56-85 years (*n* = 50)	Shotgun metagenomics	Elderly: Higher α-diversity. Significant depletion of *C. acnes*; enrichment of *Corynebacterium kroppenstedtii*, *Streptococcus*, *Staphylococcus*, and *Proteobacteria* Younger: High proportions of *C. acnes* and *Lactobacillus*, most notably *Lactobacillus crispatus*	Younger microbiome exhibits higher expression of genes related to active metabolism and innate microbial protection	[41]
Cheeks	479 healthy Chinese participants	18-64 years	Shotgun metagenomics	Elderly: Higher α-diversity. Transition from C-cutotype (*Cutibacterium*-dominated, younger) to S-cutotype (*Streptococcus*-dominated, elderly)	Developed and rigorously validated a microbiome-based facial aging index (FAI) to quantify skin aging and uncover lifestyle impacts	[42]
Forehead, cheeks, back of the nose	294 healthy Chinese participants	20-35 (*n* = 74), 36-50 (*n* = 131), and 51-65 years (*n* = 89)	Shotgun metagenomics	Elderly: Identification of 58 age-associated bacterial species. Enrichment of *Moraxella*, *Chryseobacterium*, *Elizabethkingia*, and *Paracoccus* Younger: Enrichment of *C. acnes*, *Aeromicrobium choanae*, *Malassezia globosa*, and *Debaryomyces fabryi*	*C. acnes* correlates negatively with age and aging traits, whereas *Moraxella osloensis* correlates positively with aging traits	[7]
Face, forearm	59 United Kingdom participants	26.7 ± 4.45 (*n* = 30), and 72.3 ± 4.04 years (*n* = 29)	16S rRNA gene sequencing	Identification of *C. acnes* (decreased), *S. hominis* (increased), and community diversity as key biomarkers across the lifespan	Elderly networks exhibit fewer nodes and edges compared to younger networks	[43]

OTU: Operational taxonomic unit; TEWL: transepidermal water loss; NMF: natural moisturizing factor; AMP: antimicrobial peptide; TCA: tricarboxylic acid.

However, reported age-associated microbial signatures should be interpreted in the context of substantial inter-individual and methodological variability. Several host- and environment-related factors can contribute to differences in skin microbial composition between individuals and cohorts. Anatomical site is a major source of variation because differences in sebum content, moisture, pH, and other local physiological features create distinct microbial habitats^[44,45]^. Beyond site-specific effects, ethnic background^[31,44]^, personal care practices such as cosmetic use and cleansing^[46]^, and environmental exposures, including urban versus rural living conditions^[45]^, may further contribute to inter-individual and population-level differences in microbial composition. Methodological heterogeneity also represents an important source of variability. Differences in sampling procedures, DNA extraction methods, amplicon target regions, and sequencing approaches, including 16S rRNA gene sequencing versus shotgun metagenomics, can influence taxonomic detection and relative-abundance estimates^[47,48]^. Consequently, microbial features identified in individual aging cohorts should be interpreted cautiously and validated across anatomical sites and populations using standardized analytical pipelines before they can be regarded as generalized signatures of skin aging.

Despite this variability, recent integrative analyses have extended beyond simple age correlations by examining associations between specific microbial features and biophysical signs of aging. Age-associated shifts in facial microbial communities are characterized by a decline in *Cutibacterium*-dominated profiles and are associated with changes in skin physio-optical properties^[42]^. For instance, the relative abundance of *C. acnes* is negatively associated with wrinkle severity and positively linked to skin elasticity, suggesting a potential protective role for this commensal in skin aging^[49]^. In parallel, age-associated increases in microbial diversity have been negatively associated with the collagen diffusion coefficient, linking microbial restructuring to changes in collagen-related skin properties^[39]^. This predictable restructuring supports the development of microbial aging clocks. By tracking specific markers, including the depletion of *Mycoplasma, Enterobacteriaceae,* and *Pasteurellaceae*, these models can estimate chronological age with a mean error of less than four years^[36]^.

The remodeling of the skin microbiome is driven by a convergence of intrinsic physiological changes and extrinsic environmental stressors. Aging skin is primarily characterized by altered sebaceous and lipid homeostasis, pH elevation, structural changes, and immunosenescence. With advancing age, declining sebaceous activity and sebum availability are associated with reduced *Cutibacterium* abundance and altered *Malassezia* composition^[38,50]^, and may also favor the expansion of less lipid-dependent opportunistic taxa^[51]^. Concurrently, skin surface pH tends to increase modestly with age, shifting from the more acidic conditions typical of younger skin toward a relatively less acidic state^[52]^. This loss of the acid mantle has dual consequences, impairing the growth of acidophilic symbionts while enhancing the activity of pH-sensitive serine proteases such as kallikrein-5 and -7 in the epidermis. Elevated pH enhances serine protease activity and reduces lipid-processing enzyme activity, thereby promoting corneodesmosome degradation and barrier dysfunction. These changes may favor the growth and colonization of pathobionts such as *Staphylococcus aureus* (*S. aureus*)^[53]^. Aging is also accompanied by immunosenescence, including reductions in Langerhans cell number and migratory capacity, impaired antigen-specific responses, and broader alterations in skin-resident immune populations^[54]^. These changes weaken cutaneous barrier immunity and may increase susceptibility to microbial colonization and infection. Recent Mendelian randomization studies provide genetic evidence supporting potential causal relationships between these microbial shifts and skin aging phenotypes. These findings suggest that microbial alterations may contribute to the aging process rather than merely accompany age-related changes, with *Pseudomonadales* showing a positive association with facial aging^[55,56]^.

Taken together, the skin microbiome is a dynamic and functionally active ecosystem that undergoes systematic and predictable changes with aging. These alterations, characterized by taxonomic shifts, functional dysregulation, and decreased ecological stability, suggest that the microbiome may serve as a biomarker for biological aging and provide a rationale for developing targeted microbiome-modulation strategies.

## BIDIRECTIONAL INTERACTIONS BETWEEN THE SKIN MICROBIOME AND SKIN AGING

Current evidence supports a bidirectional association between age-related changes in the skin and alterations in the skin microbiome, with experimental studies further implicating specific microbial factors in aging-related skin phenotypes. Several molecular pathways have been proposed to link the functional decline of protective symbionts and the emergence of pathogenic traits with tissue degeneration [Figure 1].

**Figure 1:**
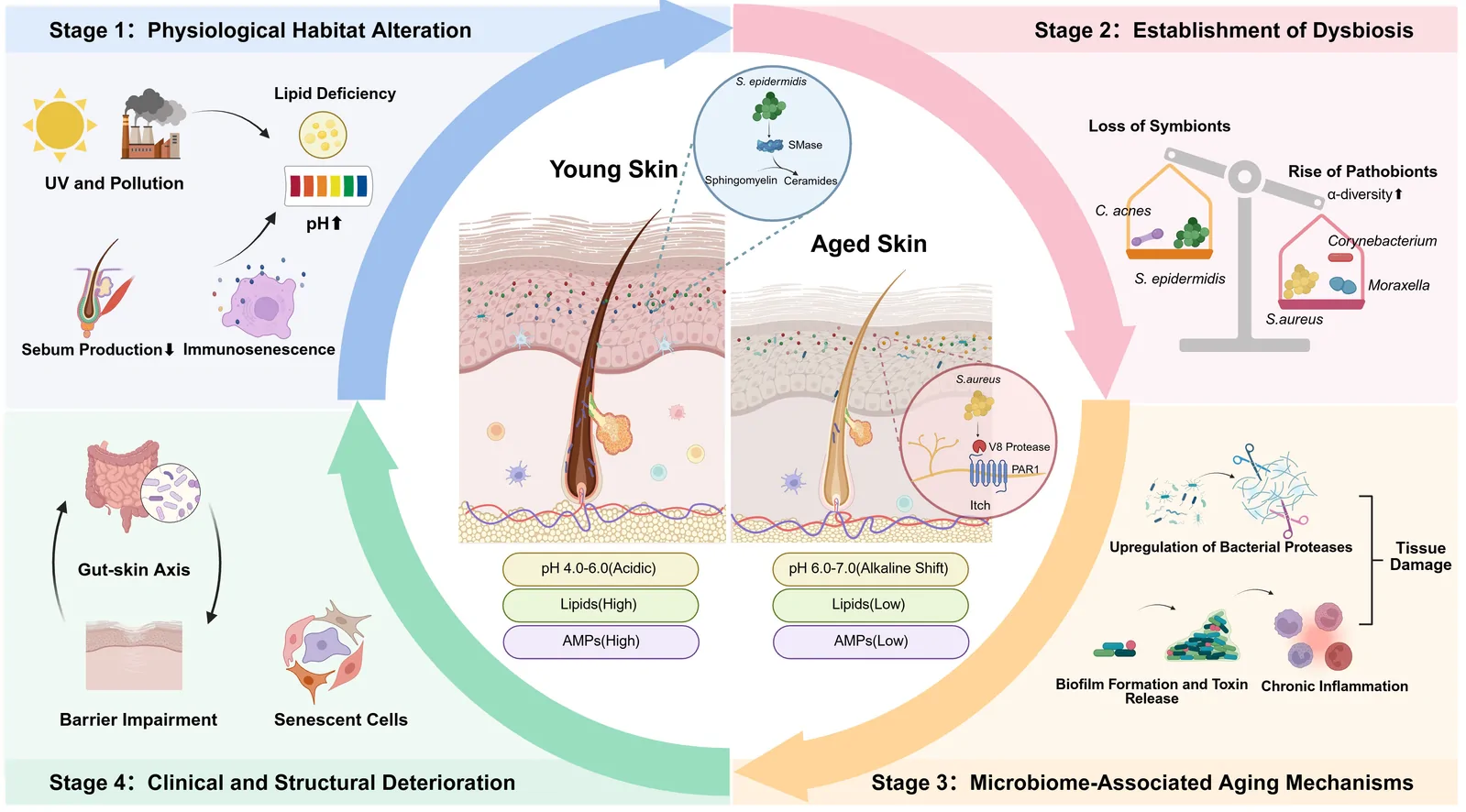
Bidirectional cycle of interactions between skin aging and microbiome dysbiosis. Intrinsic physiological decline such as reduced sebum secretion and immunosenescence synergizes with extrinsic exposome stressors like UV and pollution to reshape the cutaneous microenvironment. These factors induce an alkaline shift and lipid deficiency, favoring a pathological niche shift marked by a paradoxical increase in α-diversity, the depletion of keystone symbionts, and the proliferation of opportunistic pathobionts. This dysbiotic state is linked to tissue degeneration through specific molecular pathways, wherein bacterial proteases degrade extracellular matrix components while biofilm formation and toxin release trigger sustained chronic inflammation known as inflammaging. The resulting barrier impairment and cellular senescence further weaken host defenses to establish a self-reinforcing cycle, which may be further exacerbated by systemic signals from the gut-skin axis. UV: ultraviolet; *C. acnes*: *Cutibacterium acnes*; *S. aureus*: *Staphylococcus aureus*; *S. epidermidis*: *Staphylococcus epidermidis*; SMase: sphingomyelinase; AMP: antimicrobial peptide; PAR1: proteinase-activated receptor 1. Created in BioRender. Yu, L. (2026) https://BioRender.com/2u39vc7.

The structural integrity of the skin relies on the extracellular matrix (ECM), primarily composed of collagen and elastin. While aging involves the upregulation of host matrix metalloproteinases (MMPs), the microbiome provides an additional source of proteolytic stress. Certain cutaneous bacteria possess proteolytic capabilities that can compromise the dermal matrix. Although such activity is typically linked to wound infections, extracellular bacterial proteases produced by cutaneous bacteria such as *Staphylococcus* and *Pseudomonas* species can degrade tissue components and may contribute to age-associated alterations in skin elasticity^[57,58]^. Under dysbiotic conditions, this proteolytic activity also affects the epithelial barrier. Commensal bacteria such as *Staphylococcus epidermidis* (*S. epidermidis*) secrete the cysteine protease EcpA. Although the enzyme is largely inactive during skin homeostasis, EcpA expression increases in dysbiotic environments. Once activated, it degrades epidermal barrier proteins, including desmoglein-1 and the antimicrobial peptide LL-37. This degradation impairs barrier integrity, increases transepidermal water loss, and promotes local inflammation^[59]^.

Oxidative stress is a primary driver of skin aging. *C. acnes* plays a dual role in this process, depending on its phylotype and the local microenvironment. Under homeostatic conditions, *C. acnes* secretes RoxP, an antioxidant enzyme that protects human keratinocytes and monocytes from oxidative damage and exhibits antioxidant activity comparable to vitamin C^[60,61]^. The age-related decline in *C. acnes* abundance may therefore reduce this microbial antioxidant shield, leaving the skin more susceptible to reactive oxygen species (ROS). Conversely, specific *C. acnes* strains produce high levels of porphyrins. Upon UV exposure, these bacterial porphyrins catalyze the generation of singlet oxygen, which may exacerbate squalene oxidation and trigger an inflammatory cascade^[62]^.

In addition to direct damage, aging skin shows a deficiency in structural lipids. The commensal *S. epidermidis* secretes a specific sphingomyelinase that converts sphingomyelin into ceramides, key lipid constituents of the stratum corneum barrier^[63]^. This microbial contribution supports skin barrier integrity. In addition, metabolomic studies show that UV exposure induces microbiome-dependent alterations in skin metabolites and membrane lipids, including choline, phosphatidylcholine, phosphatidylethanolamine, and sphingomyelin^[64]^.

Chronic, low-grade systemic inflammation is a hallmark of aging and is accompanied by alterations in the cutaneous microbial ecosystem. Age-associated increases in skin pH, from approximately 6.0 to 7.0, can impair colonization resistance and create conditions that favor the expansion of potentially pathogenic taxa, thereby promoting sustained local immune activation. Recent cohort studies have identified *Corynebacterium kroppenstedtii* (*C. kroppenstedtii*) as an age-associated taxon whose abundance increases in older skin^[41,43]^. Older adults are particularly susceptible to *S. aureus*-associated skin infections due to age-related barrier and immune dysfunction. Under dysbiotic conditions, *S. aureus* can form biofilms that promote persistent colonization and local inflammation^[65]^. A primary mechanism of *S. aureus*-induced damage involves the secretion of the serine protease V8. This enzyme directly cleaves proteinase-activated receptor 1 (PAR1) on sensory neurons, driving spontaneous itch and alloknesis^[66]^. Subsequent scratching further disrupts the epidermal barrier and induces keratinocytes to release alarmins such as IL-33, thereby reinforcing a vicious cycle of itch, barrier damage, and inflammation^[67]^.

While bacterial communities are the primary focus of current research, the rare biosphere, which comprises fungi and mites, also plays a significant role. *Malassezia* yeasts are lipid-dependent commensals, and reduced skin lipid levels with age have been associated with decreased *Malassezia* abundance. To acquire essential lipids, *Malassezia* increases the secretion of lipases and phospholipases to extract fatty acids directly from host cell membranes^[68]^. This scavenging activity releases irritant-free fatty acids such as oleic acid, which can disrupt stratum corneum homeostasis and promote inflammation^[69]^. Additionally, *Demodex* abundance tends to increase with age^[70]^. Their proliferation activates the host Toll-like receptor 2 (TLR2) pathway, leading to the secretion of antimicrobial peptides, specifically cathelicidin LL-37, and pro-inflammatory cytokines^[71]^.

Finally, the relationship between the microbiome and aging extends beyond the skin surface, influencing the host systemically through the gut-skin axis. Age-associated gut dysbiosis can compromise intestinal barrier integrity and increase systemic exposure to microbial products^[72]^. Consequently, microbial components such as lipopolysaccharide (LPS) may enter the circulation and influence distal tissues, including the skin. Circulating microbial products may contribute to cutaneous inflammatory responses. Human dermal fibroblasts, for example, respond to LPS through TLR4 signaling with increased IL-8 production^[73]^. Stimulation of dermal fibroblasts with the TLR4 ligand LPS can also increase MMP-1 expression, providing a potential link between microbial inflammatory signals and ECM remodeling^[74]^. Beyond microbial structural components, gut-derived metabolites represent another important signaling layer within the gut-skin axis. SCFAs produced by commensal gut bacteria can regulate host immune responses and epithelial barrier function through receptor-mediated and epigenetic mechanisms^[75]^. Gut-derived SCFAs have also been shown to promote keratinocyte metabolism and differentiation, thereby supporting skin barrier integrity^[76]^. Similarly, microbial tryptophan metabolites have been implicated in skin barrier regulation, with the aryl hydrocarbon receptor (AhR) serving as an important pathway involved in epidermal differentiation and barrier-related gene expression. This provides a potential link between microbial metabolism and cutaneous immune homeostasis^[77,78]^. The gut-skin axis therefore acts as an active regulatory network within the holobiont system, where gut-derived signals may influence cutaneous aging phenotypes^[79]^.

## DYSBIOSIS-ASSOCIATED PATHOLOGIES IN THE ELDERLY

Age-associated dysbiosis has been implicated in the pathogenesis and progression of multiple dermatological conditions that are common in older populations. Beyond compositional shifts, microbial imbalance can impair cutaneous barrier integrity, amplify inflammatory cascades, and modify local immune surveillance. These changes create environments for disease initiation and persistence [Figure 2].

**Figure 2:**
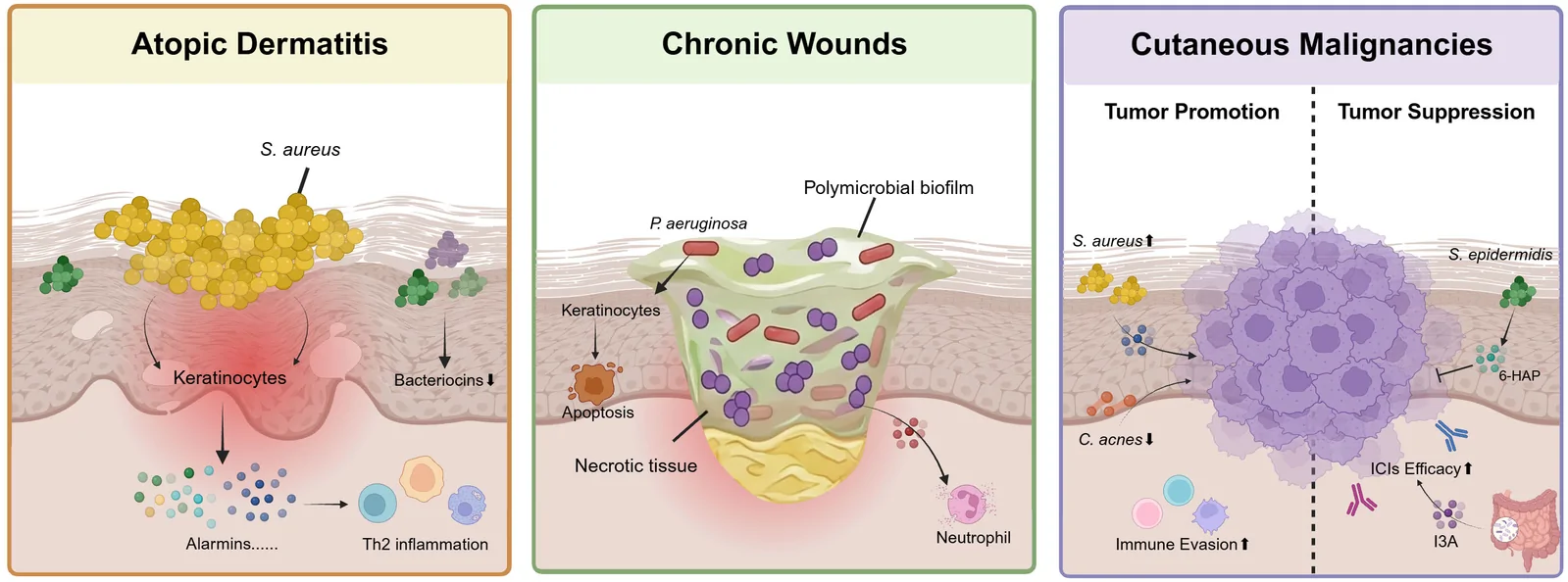
Mechanistic links between microbial dysbiosis and geriatric dermatoses. In senile atopic dermatitis, *S. aureus* virulence factors induce epithelial alarmins and drive chronic itch-scratch cycles. In chronic wounds, multispecies biofilms create a proteolytic and hypoxic environment that impairs tissue repair. In cutaneous malignancies, dysbiosis is associated with a pro-tumorigenic microenvironment characterized by inflammatory signaling and loss of protective anti-proliferative metabolites. *S. aureus*: *Staphylococcus aureus*; *P. aeruginosa*: *Pseudomonas aeruginosa*; *C. acnes*: *Cutibacterium acnes*; *S. epidermidis*: *Staphylococcus epidermidis*; Th2: T helper 2; 6-HAP: 6-N-hydroxyaminopurine; ICI: immune checkpoint inhibitor; I3A: indole-3-aldehyde. Created in BioRender. Yu, L. (2026) https://BioRender.com/tixmi0o.

In senile atopic dermatitis (AD) and chronic pruritus, the microbiome may exacerbate disease severity. While classical AD is largely driven by filaggrin mutations and Th2-mediated allergy, senile AD is primarily associated with barrier aging and immunosenescence. The microbiome in AD patients exhibits a marked reduction in diversity and a significant expansion of *S. aureus*^[80]^. The pathogenicity of *S. aureus* in senile AD is mediated through the secretion of superantigens and proteases that sustain chronic inflammation. Specifically, *staphylococcal* virulence factors induce the release of epithelial alarmins such as thymic stromal lymphopoietin and IL-33 from keratinocytes. This promotes a Th2-skewed immune response even in the absence of traditional allergens. This microbially driven inflammation exacerbates the pre-existing barrier defect and creates a cycle of pruritus and excoriation^[81]^. Furthermore, the depletion of commensal coagulase-negative *staphylococci*, such as *S. epidermidis* and *Staphylococcus hominis* (*S. hominis*), which normally produce antimicrobial peptides to control *S. aureus*, further compromises colonization resistance^[82,83]^.

Delayed wound healing is a characteristic feature of skin aging. Clinically, this manifests as venous leg ulcers, diabetic foot ulcers, and pressure injuries. These chronic wounds impose substantial healthcare burdens and markedly diminish the quality of life in older adults^[84]^. In the elderly, barrier function is compromised by reduced lipid production and epidermal thinning. This structural decline increases susceptibility to microbial colonization. Polymicrobial biofilms, which are detected in approximately 60% to 90% of chronic wound biopsies, are important contributors to chronic wound persistence. In the geriatric host, the normal transition from the inflammatory phase to the proliferative phase is impaired. This stagnation is associated with a dysbiotic community dominated by *S. aureus*, *Pseudomonas aeruginosa* (*P. aeruginosa*), and diverse anaerobes. These microorganisms reside within a self-produced extracellular polymeric matrix that shields them from host immune clearance and systemic antibiotics while facilitating metabolic cooperation^[85]^.

Microbial interactions within these biofilms are synergistic rather than static^[86]^. A large-scale analysis of 2,963 patients with chronic wounds identified *S. aureus* as a prevalent species^[87]^. Mechanistically, *S. aureus* consumes local oxygen through aerobic respiration, creating a hypoxic microenvironment that supports the proliferation of strict anaerobes. In turn, anaerobes such as *Finegoldia magna* produce proteases such as SufA, which cleaves fibrinogen and interferes with fibrin network formation^[88]^. *P. aeruginosa* further impairs healing through its type III secretion system, which delivers cytotoxic effectors such as ExoU and ExoS into host cells, thereby promoting tissue damage^[89]^. Additionally, *P. aeruginosa* produces rhamnolipids that lyse neutrophils. Under the selective pressure of the aged, often hyperglycemic wound microenvironment, this bacterium also forms hyper-biofilm variants. Collectively, the interplay between a dysbiotic, biofilm-forming microbiome and age-related host vulnerabilities can create a cycle of inflammation and tissue destruction.

Mendelian randomization studies suggest potential causal associations between specific skin microbial taxa and skin cancer risk^[90]^. Many skin cancers are strongly linked to chronic UV exposure, which not only damages DNA but also reshapes the microbial community. This pressure selects for a community of UV-tolerant microbes that may influence local immune surveillance and tumor initiation^[91]^. In squamous cell carcinoma (SCC), dysbiotic communities frequently show a reduction of *C. acnes* and an enrichment of *S. aureus*^[92,93]^. Mechanistically, *S. aureus*-derived phenol-soluble modulin α (PSMα) induces keratinocyte IL-1α and IL-36α release and drives IL-17-dependent cutaneous inflammation, providing a potential inflammatory mechanism through which *S. aureus* may influence tumor-associated immune environments^[94]^. This sustained IL-17 signaling may drive tumorigenesis by activating oncogenic STAT3 and NF-κB pathways while recruiting immunosuppressive myeloid-derived suppressor cells^[95,96]^. In basal cell carcinoma (BCC), β-human papillomavirus (β-HPV) infection has been proposed as a potential cofactor, with β-HPV DNA detected in a subset of BCC lesions and certain β-HPV types enriched relative to perilesional skin^[97,98]^. Mechanistically, E6 proteins from several β-HPV types can impair p300-dependent p53 signaling and DNA damage responses, thereby increasing the persistence of UV-induced genomic damage^[99]^.

The microbiome appears to play a complex role in melanoma pathogenesis. In this context, *S. epidermidis* exhibits a functional dichotomy. Specific strains generate 6-N-hydroxyaminopurine (6-HAP), which has been shown to suppress tumor-cell proliferation and reduce tumor growth in experimental models^[100]^. However, *S. epidermidis* can also enhance TRAF1 and CASP14 expression in melanocytes through the secretion of lipoteichoic acid. While this mechanism protects cells against UVB-induced apoptosis, it may facilitate the survival of DNA-damaged cells and thereby perpetuate genomic instability^[101]^. Conversely, *C. acnes* induces apoptosis in DNA-damaged melanocytes through coproporphyrin production and TNF-α elevation^[101]^. Tumor progression can also be accelerated by pathogens such as *Fusobacterium nucleatum* (*F. nucleatum*). More broadly, pathogens such as *F. nucleatum* can promote tumor immune evasion. Through the binding of its Fap2 protein to the inhibitory receptor TIGIT on immune effector cells, this bacterium instigates immune suppression and protects tumors from immune clearance^[102]^.

Beyond the local environment, the gut microbiome has been linked to melanoma treatment outcomes, particularly immune checkpoint inhibitor (ICI) therapy. For instance, patients with melanoma who responded to anti-PD-1 therapy exhibited a favorable gut microbiome characterized by higher diversity and enrichment of *Ruminococcaceae* and *Faecalibacterium*, a profile associated with enhanced systemic and antitumor immune responses^[103]^. Notably, *Lactobacillus reuteri* can translocate from the gut to melanoma tissues, where it produces indole-3-aldehyde (I3A), a tryptophan metabolite that locally improves anti-PD-1 responses^[104]^. In contrast, *Helicobacter pylori* (*H. pylori*) is associated with detrimental outcomes. Clinical data indicate that *H. pylori* seropositivity correlates with reduced survival in melanoma patients treated with ICIs^[105]^.

## MICROBIOME-BASED ANTI-AGING STRATEGIES

As the importance of the skin microbiome in aging becomes increasingly clear, therapeutic strategies are expanding beyond conventional barrier repair toward ecological modulation. The objective is to promote a resilient microecosystem and support functional homeostasis, rather than simply replenishing microbial abundance [Figure 3].

**Figure 3:**
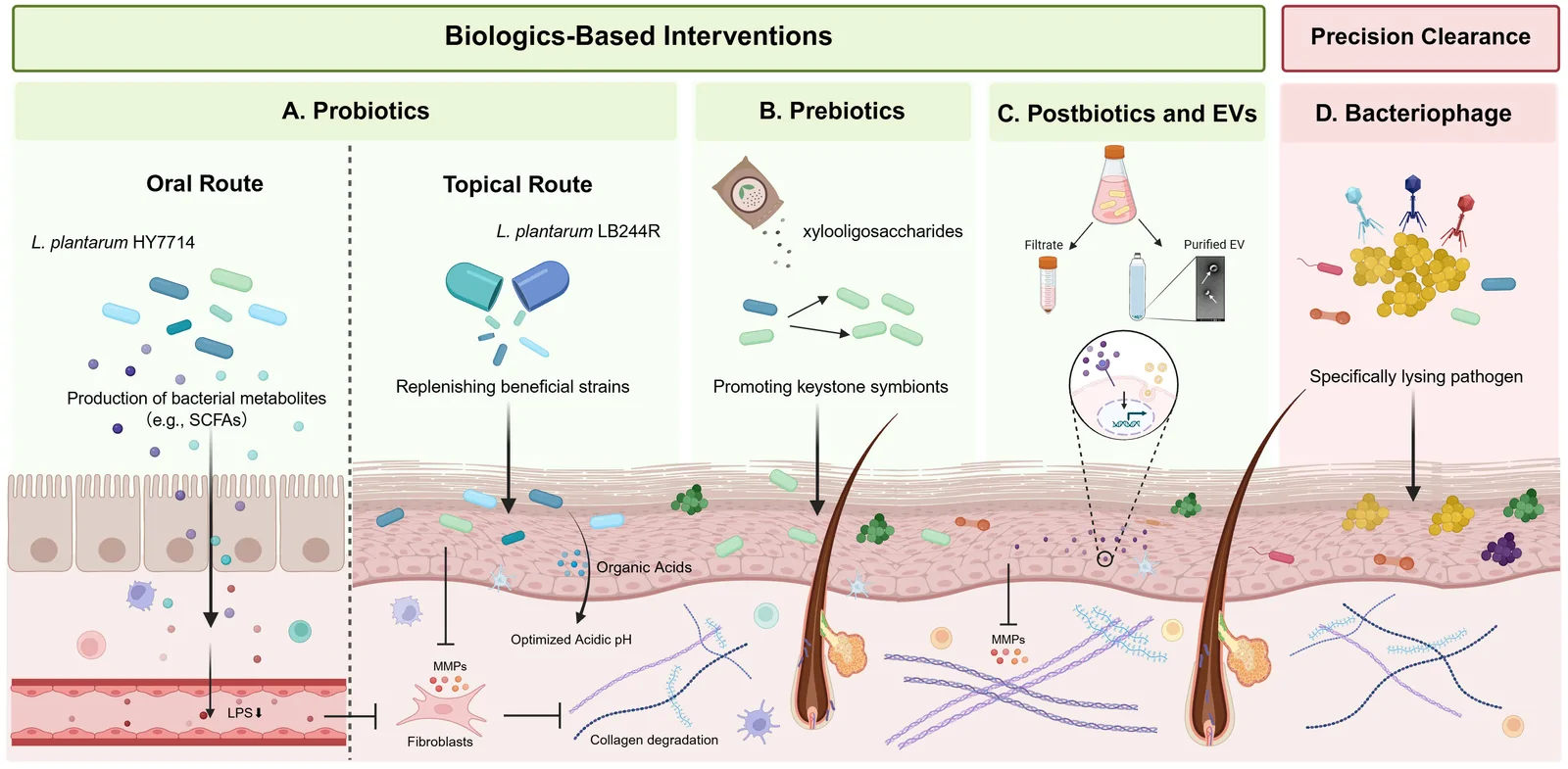
Landscape of next-generation microbiome-based therapeutic strategies. Strategies range from ecological restoration using probiotics, prebiotics, postbiotics, and bacterial EVs to optimize the microbial niche and support beneficial symbionts. Advanced precision engineering utilizes bacteriophages for the selective elimination of pathobionts to modulate microbial community structure. *L. plantarum*: *Lactiplantibacillus plantarum*; SCFA: short-chain fatty acid; MMP: matrix metalloproteinase; LPS: lipopolysaccharide; EV: extracellular vesicle. Created in BioRender. Yu, L. (2026) https://BioRender.com/6x3efls.

Biologics-based interventions form the foundation of this approach. Probiotics, defined as live microorganisms that confer a health benefit on the host, have demonstrated efficacy through both systemic and topical routes. Oral administration of *Lactiplantibacillus plantarum* (*L. plantarum*) HY7714 improved skin hydration, elasticity, wrinkle depth, and skin gloss in a randomized, double-blind, placebo-controlled trial^[106]^. Topically, the application of live *L. plantarum* LB244R has yielded positive results in older cohorts. In a double-blind, placebo-controlled trial, topical treatment significantly increased dermal density and reduced wrinkle depth. These effects may stem from the production of organic acids, such as lactic acid, which lower the local pH and inhibit colonization by opportunistic pathogens^[107,108]^. Recent advances have expanded this approach to combinatorial strategies. A randomized, double-blind trial demonstrated that the concurrent intake of a probiotic consortium containing *L. plantarum* PBS067, *Lactobacillus reuteri* PBS072, and *Lactobacillus rhamnosus LRH020*, combined with topical application of ectoin and sodium hyaluronate, significantly mitigated wrinkle depth and enhanced skin radiance. This dual-route strategy may provide complementary effects by targeting systemic and local determinants of skin health^[109]^. Complementing these live therapeutics, prebiotics offer another strategy to selectively nourish the aging skin ecosystem. Specific oligosaccharides have been proposed to selectively promote commensals such as *S. epidermidis* while suppressing potential pathogens such as *S. aureus*^[110]^.

To circumvent the stability and safety challenges associated with live bacteria, postbiotics have emerged as a cell-free alternative for anti-aging applications^[111]^. These bioactive compounds, including cell lysates, fermentation filtrates, and specific metabolites, can modulate host responses through diverse molecular mechanisms. For instance, topical application of *Epidermidibacterium keratini* (EPI-7) fermentation filtrate improved skin barrier function, elasticity, and dermal density in a randomized split-face clinical study^[112]^. Similarly, Wang et al. characterized a mixed fermentation extract (TBFE) derived from *Thermus thermophilus* and *Bacillus subtilis*. In vitro, TBFE increased type IV collagen and elastin expression, promoted autophagy, and reduced UVB-induced ROS and IL-6 production. In a clinical study, topical TBFE reduced wrinkles and improved moisturization and skin tone^[113]^. Bacterial extracellular vesicles (EVs) represent a further advance in cell-free therapy with lower immunogenicity than whole bacteria. Unlike crude lysates, these nano-sized lipid bilayer structures may allow for deep penetration through the aged stratum corneum. EVs derived from *L. plantarum* have shown potential to deliver bioactive cargoes that downregulate MMP expression^[114]^.

For conditions driven by the overgrowth of specific pathobionts, such as *S. aureus* in senile AD, bacteriophage therapy offers a precision tool for ecological engineering. Lytic phages can selectively target *S. aureus* while sparing commensals such as *S. epidermidis*, and topical phage treatment has reduced *S. aureus* burden and associated inflammation in preclinical models^[115,116]^. In parallel, phage-derived endolysins provide a cell-free alternative; the *S. aureus*-targeted endolysin XZ.700 selectively depleted *S. aureus*, restored microbial diversity, and promoted wound repair in preclinical skin models^[115,117]^. Targeted exclusion strategies that exploit commensal interactions offer a distinct mechanism. *S. hominis* A9 (ShA9), a commensal strain isolated from healthy skin, produces lantibiotics that directly inhibit *S. aureus*^[118]^. In addition, ShA9-derived autoinducing peptide (AIP) can interfere with *S. aureus* quorum sensing and suppress expression of the pro-inflammatory toxin PSMα^[119]^. These complementary mechanisms illustrate how selected commensal strains may be leveraged to precisely modulate pathogenic members of the skin microbiome.

Despite encouraging preclinical and clinical findings, several practical challenges currently limit the translation of microbiome-based interventions into routine management of skin aging. For live biotherapeutic products, manufacturing requires stringent control of strain identity, purity, viability, potency, stability, and lot-to-lot consistency, with analytical validation becoming increasingly complex for multi-strain formulations. Regulatory requirements for these products also remain incompletely harmonized across jurisdictions^[120,121]^. Microbiome transplantation presents distinct challenges. Human skin-transfer studies have shown that engraftment depends strongly on the baseline recipient microbiome, donor composition, and bacterial dose^[122]^. Fecal microbiota transplantation has also entered clinical investigation for AD, supporting the therapeutic relevance of the gut-skin axis, but such products require rigorous donor screening, standardized processing, and manufacturing controls to minimize transmissible-agent risk and product variability^[123,124]^. Personalized microbiome therapies further require validated biomarkers and standardized profiling methods to identify patient-specific microbial deficits and predict treatment responses^[125]^. Individually tailored strain combinations may further complicate product specification, potency testing, manufacturing scalability, and regulatory approval^[126]^. Collectively, these challenges underscore the need for standardized quality-control procedures, longitudinal safety evaluation, and clearer regulatory frameworks before microbiome-based therapies can be broadly implemented in skin-aging management.

Looking forward, skin microbiome transplantation represents a more comprehensive approach to resetting the aged ecological network using young, healthy donor communities^[127]^. However, its clinical viability is hindered by the priority effect, in which resident communities resist displacement. Consequently, successful implementation requires rigorous decolonization protocols and stringent safety validation to ensure stable engraftment. Ultimately, integrating multi-omics profiling with machine learning algorithms may enable personalized microbiome care. Quantifying individual microbial deficits could enable future interventions to deliver tailored probiotic cocktails or precision prebiotics, representing the next generation of precision medicine for healthy aging.

## CONCLUSION

Research on the skin microbiome offers a new perspective on skin aging, suggesting a shift from treating surface symptoms toward managing the underlying microbial ecosystem. However, translating these ecological insights into clinical practice remains a significant challenge. A fundamental obstacle is defining what constitutes a healthy skin microbiome, given the profound variation dictated by genetics, geography, and lifestyle^[128]^. Technical limitations also remain. While 16S rRNA gene sequencing provides taxonomic insights, it fails to resolve functional dynamics at the strain level. Even metagenomic sequencing is often limited by unmapped reads, highlighting the need for expanded reference databases to uncover the roles of the rare biosphere^[39,129]^. Furthermore, the field must progress from correlation toward establishing causal relationships between microbial alterations and skin aging. This will require rigorous validation through germ-free animal models, colonization trials, and longitudinal cohort studies to determine whether specific microbial shifts are true drivers of aging rather than mere passengers^[130]^.

Future research should shift from descriptive ecology to functional engineering. By combining metagenomics with metatranscriptomics, metaproteomics, and metabolomics, researchers can map the dynamic interactome between the host genome, microbiome, and exposome. AI will be important for analyzing these massive datasets to identify novel biomarkers or functional pathways linked to healthy aging^[131]^. In parallel, adopting a life-course approach is necessary to understand how an individual’s microbial capacity develops from infancy to old age. This perspective allows interventions that support healthy aging across the lifespan rather than only in later stages. There is also potential in identifying microbial metabolites with antioxidant or collagen-stimulating properties. Finally, developing strategies that target the gut-skin axis offers a practical way to manage systemic inflammation and improve skin health^[111]^.

In conclusion, integrating microbiome science into the study of skin aging represents a conceptual transition from a host-centric model toward a holistic holobiont perspective. The skin microbiome is increasingly recognized not as a passive bystander but as an integral component of skin physiology that may influence aging-related skin phenotypes. Understanding these host-microbe interactions provides a foundation for developing precision ecological modulation strategies to support long-term skin health.

## DECLARATIONS

### AI and AI-assisted tools Statement

During the preparation of this manuscript, the AI tools Gemini (Google, Gemini 3 Flash, released 2025-12-17) and ChatGPT (OpenAI, GPT-5.6 Sol, released 2026-07-09) were used solely for language editing. These tools did not influence the study design, data collection, analysis, interpretation, or the scientific content of the work. All authors take full responsibility for the accuracy, integrity, and final content of the manuscript.

### Copyright

© The Author(s) 2026.
